# Are flowable composite resins containing bioactive particles truly biocompatible?

**DOI:** 10.4317/jced.63914

**Published:** 2026-05-29

**Authors:** Lara Rabelo Aragão, Julia Gonçalves Montenegro, Clarice Lioba de Araújo, José Vitor Mota Lemos, Dayrine Silveira de Paula, Sergio Lima Santiago, Suely Bezerra dos Santos, Paulo Goberlânio de Barros Silva, Jiovanne Rabelo Neri

**Affiliations:** 1 Master in Dental Sciences, University Christus, Fortaleza, Ceará, Brazil; 2 Graduate School of Dentistry, University Christus, Fortaleza, Ceará, Brazil; 3 Postgraduate Program in Dentistry, Faculty of Pharmacy, Dentistry and Nursing, Federal University of Ceará (UFC), Fortaleza, Ceará, Brazil

## Abstract

**Background:**

To evaluate the degree of conversion and biocompatibility of flowable composite resins containing bioactive particles using in vitro and in vivo models, respectively.

**Materials and Methods:**

The flowable composite resins Beautifil Flow Plus F00 (F00), Beautifil Flow Plus F03 (F03), and Filtek Supreme (FS) were tested. For the degree of conversion (DC) test, seven specimens were prepared for each group and analyzed using Raman spectroscopy. For biocompatibility (BC), seven specimens from each group were implanted into the dorsal subcutaneous tissue of 21 female Wistar rats, which were divided into three groups according to the experimental periods of 7, 14, and 28 days. After euthanasia, the dorsal tissues were surgically excised, fixed in formalin solution, and processed for histological analysis. The slides were evaluated blindly, and ten microscopic fields were randomly selected at 400× magnification. Statistical analysis for DC and BC was performed using the Kruskal-Wallis test, followed by Tukey's post hoc test for DC and Dunn's test for BC.

**Results:**

For BC, F00 and F03 induced less inflammation and granulation tissue formation and promoted greater fibrosis than FS (p &lt; 0.05). For DC, no statistically significant difference was observed among the groups (p &gt; 0.05).

**Conclusions:**

Flowable bioactive composite resins represent promising alternatives for dental practice, as they induce minimal tissue inflammation and exhibit high conversion rates.

## Introduction

Composite resins are currently the most widely used materials in clinical dental practice for direct restorations. Their popularity is mainly attributed to their ease of handling, versatility, mechanical strength, and excellent esthetics (1 , 2). However, despite these advantages, composite resins still present limitations when in direct contact with oral tissues (3). In restorative procedures involving deep cavities near the pulp or in the cervical third of teeth, where contact with periodontal tissue may occur, there is a potential risk of inflammation in adjacent vital tissues (3 , 4). These inflammatory processes may be associated with two main factors: the composition of the composite resins and the degree of conversion of monomers into polymers (5). When the degree of conversion is low, greater amounts of free monomers may be released, migrating into the surrounding tissues and inducing cytotoxic and irritative reactions in the gingiva and periodontal ligament (5 - 7). Therefore, improving composite resin formulations without compromising the degree of conversion is essential to minimize their inflammatory potential and ensure greater biological safety for patients (7 - 9). The search for more biocompatible composite resins has led the dental industry to incorporate bioactive components into restorative formulations (8). In this context, Giomer technology stands out because it is based on the use of surface pre-reacted glass (SPRG) particles (9). These particles are derived from a bioactive fluoroaluminosilicate glass containing elements such as fluoride, boron, aluminum, and silicate (10 , 11). The glass is first melted and molded into uniform, polished spheroidal particles, which are then subjected to controlled grinding (12). To enable ion release and recharge, the glass surface is coated with a porous silica protective layer (12). Subsequently, this coated particle is treated with polyacrylic acid, which penetrates the silica porosities and reacts with the surface layer of the glass, forming a functional ionic layer (12 , 13). This layer is responsible for ion exchange, enabling the release and recharge of essential ions such as sodium (Na+), which facilitates the activity of other ions; strontium (Sr²+), which neutralizes acids; fluoride (F+), which prevents demineralization; silicate (SiO3²-), which contributes to remineralization; aluminum (Al³+), which prevents hypersensitivity; and borate (BO3³-), which inhibits bacterial adhesion (12 , 13). Unlike conventional glass ionomers, whose matrix is fully reacted and has reduced mechanical resistance, SPRG particles have an unreacted glass core that provides greater hardness and strength while maintaining the mechanical properties of the composite resin into which they are incorporated. When the resin containing these particles is placed in the oral environment, contact with moisture triggers the reaction in the ionic layer, promoting controlled ion release and recharge in response to the ionic balance of the oral cavity. This enhances bioactivity and contributes to the maintenance of oral health (9 , 14). Composite resins incorporating SPRG particles, such as Beautifil Flow Plus, stand out because they combine the esthetics and mechanical strength of conventional composites with the bioactivity of glass ionomer cements (GICs). This technology has shown promise for clinical applications that require materials with high mechanical performance and preventive properties, such as inhibition of bacterial adhesion and prevention of dental demineralization (15 , 16). Thus, these bioactive resins may reduce caries recurrence and the associated costs in both public and private oral healthcare settings. Despite the considerable interest in composite resins containing SPRG bioactive particles, current evidence remains limited regarding their direct biological interaction with living tissues. Most available studies have focused on physicochemical properties, ion release, or antibacterial effects, with few integrated investigations combining polymerization efficiency and in vivo tissue response (15 , 16). In particular, it remains unclear whether the incorporation of bioactive particles can enhance biocompatibility without compromising the degree of conversion, which is a critical determinant of monomer release and biological safety. In this context, subcutaneous implantation models in rodents have been widely adopted as standardized and reproducible methods for evaluating the biocompatibility of dental materials (17 - 19). This model enables direct assessment of inflammatory response, tissue organization, and healing patterns in a controlled environment, minimizing confounding factors present in the oral cavity, such as saliva, biofilm, and mechanical stress (17 , 18). Although it does not fully replicate intraoral conditions, it provides valuable preliminary evidence regarding the biological behavior of materials when in contact with vital tissues. Considering the need for an integrated evaluation of polymerization efficiency and biological response, the present study aimed to assess the degree of conversion and biocompatibility of these resins using both in vitro and in vivo models. The null hypotheses tested were as follows: (1) composite resins containing bioactive particles do not show a significant difference in degree of conversion compared with the control group; and (2) composite resins containing bioactive particles do not show a significant difference in biocompatibility compared with the control group.

## Materials and Methods

- Sample size calculation Based on the study by Itota et al. (9), which demonstrated that resin-modified ionomers (compomers) containing bioactive glass particles exhibited greater fluoride release after four days of immersion in aqueous solution than compomers without these particles (14.99 ± 1.06 vs. 11.66 ± 2.04), it was estimated that six discs per group would be required to achieve 90% power at a 95% confidence level. Considering the possibility of sample loss during the course of the experiment, an additional 10% was included, resulting in a total of seven samples per study group. - Specimen preparation Seven specimens of each tested flowable composite resin (Table 1) were prepared using a metallic mold with a central cavity measuring 5 mm in diameter and 2 mm in height.

Table 1

**Figure 1:**
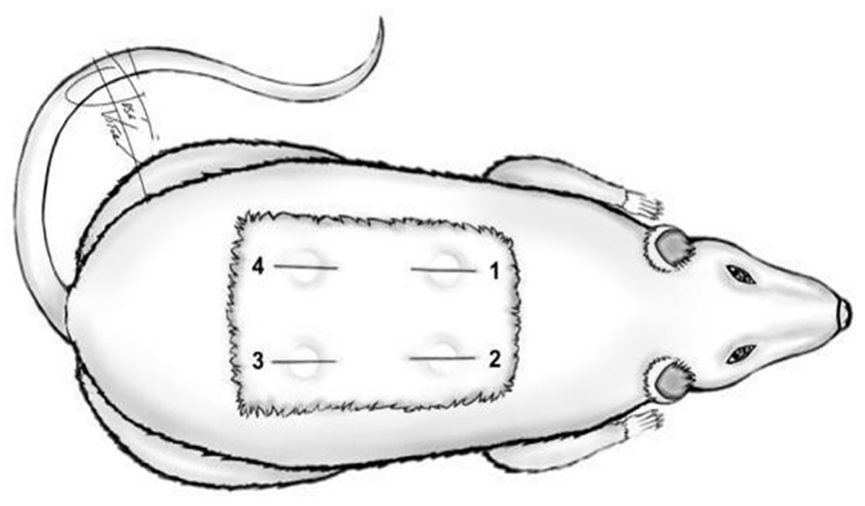
Schematic illustration of the experimental model for subcutaneous implantation of photoactivated composite resin discs in Wistar rats. Quadrant 1 = Sham; Quadrant 2 = Filtek Supreme (3M®); Quadrant 3 = Beautifil Flow Plus F03; Quadrant 4 = Beautifil Flow Plus F00.

Each specimen was prepared by injecting a single increment of composite resin into the center of the mold, avoiding the incorporation of air bubbles. The resin was then covered with a polyester strip and a glass coverslip and light-cured for 20 s at an intensity of 1000 mW/cm² (VALO, Ultradent, South Jordan, Utah, USA). After polymerization, the discs were removed by extrusion. Thus, cylindrical specimens (5 mm Ø × 2 mm) were obtained, with the top surface being directly irradiated. For each composite resin, seven specimens were prepared for the degree of conversion and biocompatibility tests. - Degree of conversion test - in vitro The degree of conversion of each specimen was determined using a micro-Raman spectrometer (Xplora; Horiba Scientific, Kyoto, Japan). The spectra were obtained with a 488 nm excitation laser through a 100× objective lens. The spectral acquisition parameters were as follows: irradiation time, 60 s; number of accumulations, 10; and grating, 1200 lines/mm. The degree of conversion was calculated based on the reduction in intensity of the methacrylate C=C stretching peaks at 1636 cm-¹ and 1608 cm-¹ in the polymerized (P) specimen compared with the unpolymerized (U) specimen, according to the following equation: Degree of conversion = (1 - P/U) × 100 - Biocompatibility test - animal model The study was approved by the Institutional Animal Ethics Committee (Protocol No. 007/21). Twenty-one female Wistar rats (Rattus norvegicus) weighing between 180 and 200 g were used in this study. The animals were randomly allocated (Microsoft Excel 2010, "Random" function; Microsoft Corporation) into three groups and maintained with food and water ad libitum under a 12-h light/dark cycle at a temperature of 20-25 °C. The position of the materials across quadrants was not randomized; instead, a fixed distribution was adopted in all animals to standardize the surgical procedure and maintain anatomical consistency among implantation sites. The dorsal region of each animal was shaved and disinfected with 2% chlorhexidine spray. Following antisepsis, the animals were anesthetized with xylazine (20 mg/kg) and ketamine (80 mg/kg) (Syntec, Tamboré, SP, Brazil) and received four 2-cm cranio-caudal incisions using a no. 15 scalpel blade mounted on a Bard Parker handle. The incisions were made in quadrants I (left anterior), II (right anterior), III (right posterior), and IV (left posterior), in a clockwise sequence starting from the left anterior quadrant. Each incision was placed 3 cm apart from edge to edge, both horizontally and vertically (Fig. 1).

1

After blunt dissection with forceps, the incision in quadrant I was sutured without tube insertion (Sham group); quadrant II was sutured with insertion of a disc from the FS group (control); and quadrants III and IV were sutured with insertion of discs from the F03 and F00 groups, respectively. The discs previously analyzed by Raman spectroscopy were stored in a protected environment away from light and, after disinfection with 2% chlorhexidine, were implanted into the dorsal subcutaneous tissue of the animals. Wound closure was performed with 4-0 nylon sutures (Procare, São Paulo, SP, Brazil) (17). - Euthanasia The animals were euthanized at 7, 14, and 28 days after the surgical procedure by overdose of xylazine (60 mg/kg) and ketamine (240 mg/kg). The dorsal tissue was surgically excised, fixed in 10% neutral buffered formalin for 24 h, and then processed for histological slide preparation (hematoxylin-eosin staining). - Histological processing After fixation, the samples were subjected to histological processing, including dehydration in graded ethanol solutions (80%, 90%, 95%, and absolute alcohol), clearing in xylene, and paraffin embedding at 65 °C using an automated tissue processor (PT09, Lupetec, São Carlos, SP, Brazil). Serial sections of 4 µm were cut using a microtome (RM 2125, Leica, Wetzlar, Germany) and mounted on frosted-edge glass slides for hematoxylin-eosin staining. After deparaffinization and rehydration, the slides were immersed in 7% Harris hematoxylin solution for 5 min, rinsed, counterstained with 10% alcoholic eosin for 10 s, dehydrated, cleared, and mounted with Entellan® coverslips. - Microscopic analysis The histological slides were blindly examined by an experienced oral pathologist using a conventional optical microscope (DM2000, Leica, Wetzlar, Germany) equipped with a digital camera for photomicrographs (DFC 295, Leica, Wetzlar, Germany). Ten microscopic fields were randomly selected at 400× magnification, and the intensity of the inflammatory infiltrate was evaluated. The infiltrate was classified according to its intensity as absent (score 0), mild (score 1; up to 25 inflammatory cells per field), moderate (score 2; 26-125 inflammatory cells per field), or severe (score 3; more than 125 inflammatory cells per field) (17). - Statistical analysis Statistical analyses were performed using SigmaStat 4.0 (Systat Software Inc., San Jose, CA, USA) and GraphPad Prism 5.0 (GraphPad Software, Boston, MA, USA). The Shapiro-Wilk and Brown-Forsythe tests were applied to all groups to assess normal distribution and homogeneity of variance, respectively. For biocompatibility analysis, histological scores were expressed as absolute and relative frequencies and compared using Pearson's chi-square test. For the degree of conversion data, one-way analysis of variance (ANOVA) was used. For biocompatibility scores expressed as median (minimum-maximum), comparisons were made using the Kruskal-Wallis test followed by Dunn's post hoc test. In all analyses, the significance level was set at p &lt; 0.05.

## Results

Degree of conversion: Addition of SPRG particles did not interfere with the degree of conversion of flowable resins The data regarding the degree of conversion for each group are presented in Table 2.

Table 2

**Figure 2:**
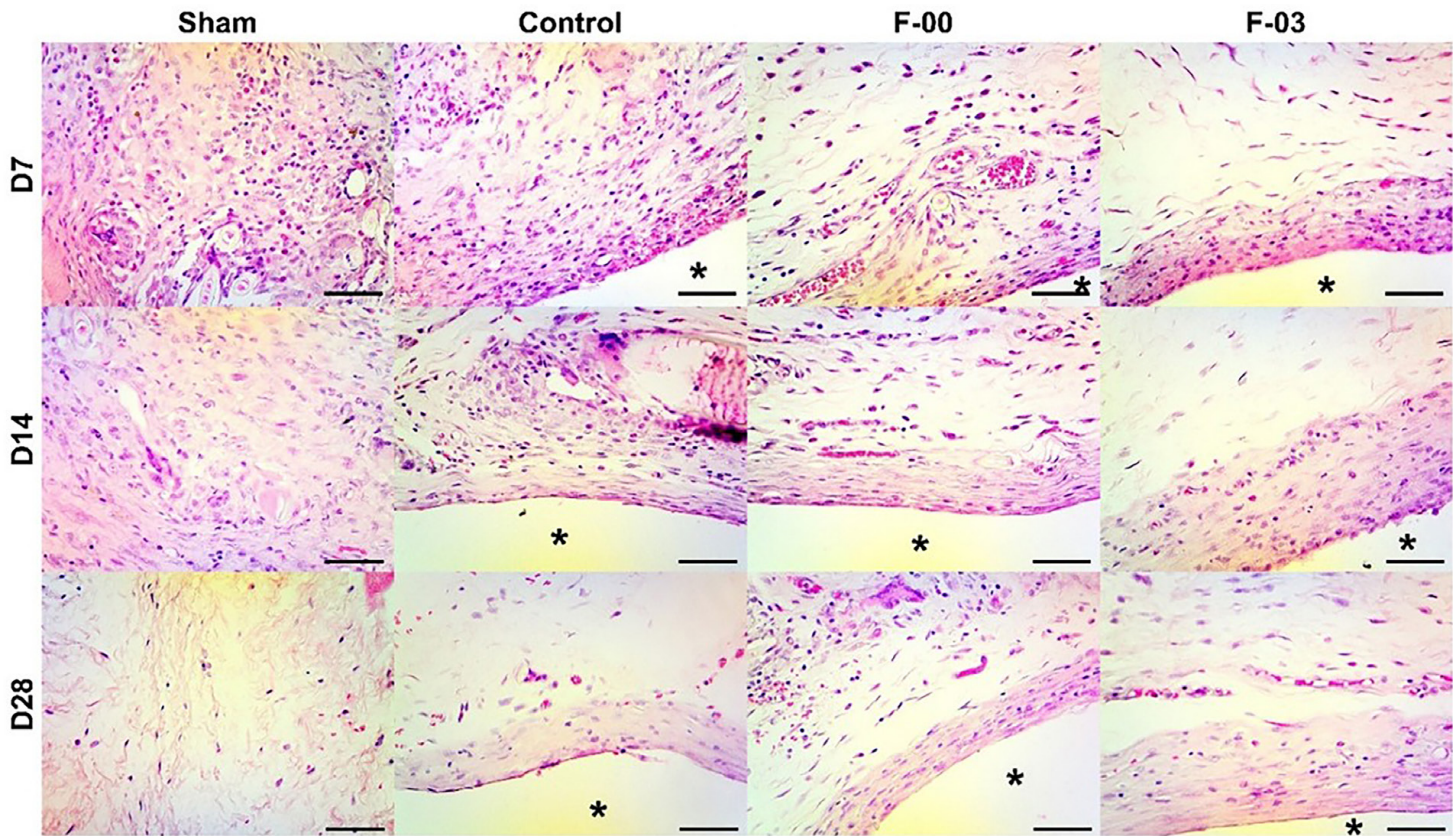
Histological analysis of the inflammatory process.

No statistically significant differences were observed among the groups (p &gt; 0.05). Histological analysis of the inflammatory process: Flowable composite resins containing SPRG particles induced lower inflammation The histological data for each group are presented in Table 3 and Figure 2.

Table 3

2

Seven days after subcutaneous implantation of the resin discs, all groups exhibited a predominantly chronic inflammatory profile, with no statistically significant differences among them (p = 0.536). The intensity of the inflammatory process was similar among the groups, with scores ranging from moderate to severe. Although no statistically significant differences were found among the groups (p = 0.561), the F03 resin showed the highest proportion of animals with moderate inflammation intensity (Fig. 2). After 14 days, the Sham, control, and F00 groups still exhibited a high frequency of a predominantly chronic inflammatory process, whereas all animals in the F03 group showed absence of inflammation (p = 0.013). The intensity of inflammation also differed significantly among the groups: the Sham and control groups presented a moderate inflammatory response, the F00 group exhibited a mild inflammatory response, and the F03 group showed no signs of inflammation (p = 0.041) (Fig. 2). After 28 days, only one animal in the F00 group exhibited a mild inflammatory process adjacent to the resin disc. However, most or all specimens in the other groups no longer showed inflammation. No differences were observed in either the profile (p = 0.372) or the intensity (p = 0.372) of the inflammatory process among the groups (Fig. 2). The Sham (p = 0.010), FS (p = 0.002), F00 (p = 0.012), and F03 (p &lt; 0.001) groups all exhibited changes in the inflammatory profile by day 14. Regarding the intensity of inflammation, a significant reduction from severe to moderate was observed in the Sham (p = 0.003), FS (p = 0.008), and F00 (p = 0.011) groups between days 7 and 14. In contrast, in the F03 group, the intensity significantly decreased from moderate to mild (p = 0.001) (Fig. 2). Histological analysis of the inflammatory process: Flowable composite resins containing SPRG particles reduced granulation tissue formation and accelerated fibrosis At day 7 after resin disc implantation, granulation tissue appeared thick in all groups except the F03 group, which showed a higher frequency of thin granulation tissue than the others (p = 0.015). After 14 days, most specimens exhibited either mild or absent granulation tissue, and although no significant differences were found among the groups (p = 0.180), the F03 group showed complete absence of granulation tissue in all specimens. By day 28, no signs of granulation tissue were observed in any group. The thickness or area of granulation tissue significantly decreased from thick to thin between days 7 and 14 in the Sham (p &lt; 0.001), FS (p &lt; 0.001), and F00 (p = 0.010) groups. In contrast, in the F03 group, granulation tissue significantly decreased from thin to absent during the same period (p = 0.001). Signs of fibrosis surrounding the disc were absent in nearly all animals in the Sham and FS groups. However, the F00 and F03 groups already exhibited predominantly thin fibrous capsules around the disc (p = 0.009). After 14 days, no difference was observed among the groups. Most animals already exhibited a thin fibrous capsule around the disc at 14 days (p = 0.472). By 28 days, all specimens from animals with implanted discs presented fibrous capsules, either thin or thick, with values significantly higher than those observed in the Sham group (p &lt; 0.001). In the Sham group, the presence of fibrosis increased from day 7 to day 14 and significantly decreased by day 28 (p &lt; 0.001). In the FS group, fibrosis significantly increased from day 7 to day 14 and continued to increase through day 28 (p &lt; 0.001). In the F00 (p = 0.371) and F03 (p = 0.354) groups, fibrosis appeared early, at day 7, and did not show significant variation thereafter. After 7 days, no specimen exhibited endocytosed material (p p = 1.000). Although no significant differences were observed among the groups at this time point (p = 0.091), after 14 days, two specimens (28.6%) from the F03 group already showed endocytosed material. By 28 days, two-thirds of the specimens in the F00 and F03 groups presented macrophages with endocytosed material in their cytoplasm, values significantly higher than those observed in the Sham and FS groups (p = 0.007). The frequency of endocytosed material significantly increased in the F00 (p = 0.004) and F03 (p &lt; 0.001) groups by day 28 after specimen implantation.

## Discussion

This study evaluated the biocompatibility and degree of conversion of flowable composite resins containing bioactive particles using both in vitro analyses and an animal model. The incorporation of these particles did not affect the degree of conversion, as evidenced by the absence of statistically significant differences among the groups, thereby confirming the first null hypothesis. In contrast, the biological tissue response improved over time, particularly in the F03 group. These findings highlight the potential clinical relevance of bioactive resins, especially in restorative procedures involving close interaction with soft tissues, such as cervical restorations or deep cavities near the pulp. The biocompatibility of flowable resin composites is directly related to their degree of conversion, since unpolymerized monomers may be released into the oral environment and exert cytotoxic effects on oral tissues (20). The lack of differences in degree of conversion among the groups is consistent with the findings of Jäger et al. (21), who reported that the incorporation of bioactive particles (BG 45S5) did not affect the degree of conversion of experimental composite resins. The study by Itota et al. (9) reported that glass ionomer-modified resins released less fluoride than resins containing bioactive particles after four days of immersion in aqueous solution (14.99 ± 1.06 vs. 11.66 ± 2.04) (7), supporting the high ion-release capacity of resins employing SPRG technology. In the present study, at 7 days, the F03 group, which has a greater potential for fluoride release, showed the highest proportion of moderate inflammation. This response may be associated with the initial phase of ion release, which tends to stabilize over time. However, this mechanism remains hypothetical and was not directly investigated in the present study. At 14 days, the F03 group showed distinct behavior compared with the other groups, being the only group without signs of inflammation. In contrast, the Sham and FS groups still exhibited a moderate inflammatory response, while the F00 group showed a mild response. This finding led to rejection of the second null hypothesis. These results are consistent with the hypothesis that ions released from SPRG particles may contribute to a more stable and less reactive tissue microenvironment. By 28 days, all groups exhibited either no inflammation or only mild traces, indicating the transient nature of the initial inflammatory response. The F03 and F00 groups also showed reduced granulation tissue formation and earlier fibrosis. At day 7, the F03 group exhibited predominantly thin granulation tissue, whereas the other groups showed thicker tissue. After 14 days, granulation tissue was absent in the F03 group but still present in the others. The early presence of thin fibrous tissue surrounding the discs in the bioactive groups may indicate a more organized reparative response. However, this finding should be interpreted cautiously, as fibrosis may also represent a foreign body reaction depending on the biological context. These effects may be related to the modulatory action of ions released from SPRG particles on cellular pathways associated with inflammation and tissue repair (22). These findings are consistent with previous studies indicating that materials containing SPRG particles can positively influence tissue response, promoting healing and stability in the surrounding environment. Evidence from studies on endodontic cements suggests that these materials may modulate signaling pathways such as MAPK and ERK, thereby promoting cell differentiation and tissue repair (22). In the present study, the higher frequency of endocytosed material observed in the F00 and F03 groups after 28 days further suggests increased phagocytic activity associated with the bioactivity of these materials. This study contributes to the literature by evaluating both the biocompatibility and degree of conversion of flowable composite resins containing SPRG particles using combined in vitro and in vivo approaches. These materials promoted a more favorable tissue response, characterized by reduced granulation tissue formation, earlier fibrosis, and a more controlled inflammatory process, without compromising the degree of conversion. However, some limitations should be considered, including the use of a subcutaneous model that does not fully reproduce the oral environment, the 28-day evaluation period, the limited number of formulations tested, and the absence of complementary cytotoxicity and ion-release analyses. Further long-term clinical studies with a broader range of materials and additional functional assessments are needed to confirm these findings. Another limitation relates to the sample size calculation, which was based on data from studies evaluating bioactive materials with similar properties rather than on the primary outcomes of this study. This approach was necessary due to the lack of studies simultaneously assessing degree of conversion and in vivo biocompatibility of flowable composite resins containing SPRG particles and should therefore be interpreted as an approximation. Nevertheless, the sample size is consistent with previous in vivo studies using subcutaneous implantation models and was sufficient to detect statistically significant differences in relevant biological parameters. Future studies with larger sample sizes and calculations based on directly comparable outcomes are recommended.

## Conclusions

Flowable composite resins containing SPRG bioactive particles demonstrated favorable biocompatibility in a subcutaneous model, with reduced inflammatory response and indications of accelerated tissue repair, particularly for Beautifil Flow Plus F03. The presence of these particles did not compromise the degree of conversion of the resins, suggesting that their bioactive effects are associated with the properties of the incorporated particles rather than with the polymerization efficiency of the material. These findings highlight the potential of such materials as bioactive restorative alternatives; however, further studies are required to confirm their long-term biological behavior and clinical applicability under intraoral conditions.

## Figures and Tables

**Table 1:** Table Composition of the composite resins tested in the study.

Composite Resins	Composition	Filler content (wt%)	Shade	Flowability	Manufacturer
Filtek Supreme (FS)	BIS-GMA, UDMA, TEGDMA, HEMA, Silica, Zirconia	65.0%	A3E	High	3M ESPE Dental Products, St. Paul, MN, USA
Beautifil Flow Plus F00 (F00)	Bis-GMA, TEGDMA, photoinitiator, Silica, SPRG fillersG	67.3%	A3E	Low	SHOFU INC., Fukuine, Higashiyama-ku, Kyoto, Japan
Beautifil Flow Plus F03 (F03)	Bis-GMA, TEGDMA, photoinitiator, Silica, SPRG fillers	66.8%	A3E	High	SHOFU INC., Fukuine, Higashiyama-ku, Kyoto, Japan

1

**Table 2:** Table In vitro degree of conversion values (mean ± standard deviation) according to the flowable composite resin used.

Groups (n = 7)	Degree of Conversion
FILTEK Supreme (FS)	75.20 ± 1.67A
BEAUTIFIL FLOW PLUS F00 (F00)	73.66 ± 1.39A
BEAUTIFIL FLOW PLUS F03 (F03)	75.04 ± 1.96A

*Identical uppercase letters indicate no statistically significant difference in the columns.

**Table 3:** Table Cellular and inflammatory profile in the dorsal subcutaneous tissue of rats following implantation of different resin discs.

	7 days				PValue	14 days				pValue	28 days				p-Value
	Sham	FS	F00	F03		Sham	FS	F00	F03		Sham	FS	F00	F03	
Inflammatory Profile															
Absent	0(0.0%)	0(0.0%)	0(0.0%)	0(0.0%)	0.536	3(42.9%)	1(14.3%)	4(57.1%)	7(100.0%)*	0.013	6(100.0%)	6(100.0%)	5(83.3%)	6(100.0%)	0.372
Acute	1(16.7%)	1(16.7%)	0(0.0%)	0(0.0%)		0(0.0%)	0(0.0%)	0(0.0%)	0(0.0%)		0(0.0%)	0(0.0%)	0(0.0%)	0(0.0%)	
Chronic	5(83.3%)	5(83.3%)	6(100.0%)	6(100.0%)		4(57.1%)*	6(85.7%)*	3(42.9%)*	0(0.0%)		0(0.0%)	0(0.0%)	1(16.7%)	0(0.0%)	
Inflammation Score															
Absent	0(0.0%)	0(0.0%)	0(0.0%)	0(0.0%)	0.561	3(42.9%)	3(42.9%)	4(57.1%)	7(100.0%)*	0.041	6(100.0%)	6(100.0%)	5(83.3%)	6(100.0%)	0.372
1-25 cells /field	0(0.0%)	0(0.0%)	0(0.0%)	0(0.0%)		0(0.0%)	1(14.3%)	2(28.6%)*	0(0.0%)		0(0.0%)	0(0.0%)	1(16.7%)	0(0.0%)	
26-125 cells/field	3(50.0%)	3(50.0%)	3(50.0%)	5(83.3%)		4(57.1%)*	3(42.9%)*	1(14.3%)	0(0.0%)		0(0.0%)	0(0.0%)	0(0.0%)	0(0.0%)	
> 125 cells /field	3(50.0%)	3(50.0%)	3(50.0%)	1(16.7%)		0(0.0%)	0(0.0%)	0(0.0%)	0(0.0%)		0(0.0%)	0(0.0%)	0(0.0%)	0(0.0%)	
Granulation Tissue															
Absent	0(0.0%)	0(0.0%)	0(0.0%)	0(0.0%)	0.015	4(57.1%)	3(42.9%)	4(57.1%)	7(100.0%)	0.180	6(100.0%)	6(100.0%)	6(100.0%)	6(100.0%)	1.000
Thin	0(0.0%)	1(16.7%)	2(33.3%)	5(83.3%)*		3(42.9%)	4(57.1%)	2(28.6%)	0(0.0%)		0(0.0%)	0(0.0%)	0(0.0%)	0(0.0%)	
Thick	6(100.0%)*	5(83.3%)*	4(66.7%)*	1(16.7%)		0(0.0%)	0(0.0%)	1(14.3%)	0(0.0%)		0 (0.0%)	0(0.0%)	0(0.0%)	0(0.0%)	
Fibrosis / Fibrous Capsule															
Absent	6(100.0%)*	5(83.3%)*	2 (33.3%)	1 (16.7%)	0.009	1(14.3%)	0(0.0%)	1(14.3%)	1(14.3%)	0.472	6(100.0%)*	0(0.0%)	0(0.0%)	0(0.0%)	<0.001
Thin	0(0.0%)	1(16.7%)	4 (66.7%)*	5 (83.3%)*		6(85.7%)	7(100.0%)	5(71.4%)	4(57.1%)		0(0.0%)	3(50.0%)*	4(66.7%)*	3(50.0%)*	
Thick	0(0.0%)	0(0.0%)	0 (0.0%)	0 (0.0%)		0(0.0%)	0(0.0%)	1(14.3%)	2(28.6%)		0(0.0%)	3(50.0%)*	2(33.3%)*	3(50.0%)*	
Endocytosed material															
Absent	6(100.0%)	6(100.0%)	6(100.0%)	6(100.0%)	1.000	7(100.0%)	7(100.0%)	7(100.0%)	5(71.4%)	0.091	6(100.0%)*	6(100.0%)*	2(33.3%)	2(33.3%)	0.007
Present	0(0.0%)	0(0.0%)	0(0.0%)	0(0.0%)		0(0.0%)	0(0.0%)	0(0.0%)	2(28.6%)		0(0.0%)	0 (0.0%)	4(66.7%)*	4(66.7%)*	

3

## Data Availability

The datasets used and/or analyzed during the current study are available from the corresponding author.
